# Acoustography by Beam Engineering and Acoustic Control Node: BEACON

**DOI:** 10.1002/advs.202403742

**Published:** 2024-10-18

**Authors:** Wenjun Yu, Haodong Zhu, Neil Upreti, Brandon Lu, Xianchen Xu, Luke P Lee, Tony Jun Huang

**Affiliations:** ^1^ Department of Mechanical Engineering and Material Science Duke University Durham NC 27708 USA; ^2^ Department of Biomedical Engineering Duke University Durham NC 27708 USA; ^3^ Harvard Medical School Division of Engineering in Medicine Department of Medicine Brigham and Women's Hospital Harvard University Boston MA 02115 USA; ^4^ Department of Bioengineering Department of Electrical Engineering and Computer Science University of California Berkeley CA 94720 USA; ^5^ Institute of Quantum Biophysics Department of Biophysics Sungkyunkwan University Suwon South Korea 16419

**Keywords:** acoustic manipulation, acoustofluidics, orbital angular momentum (OAM) beam

## Abstract

Acoustic manipulation has emerged as a valuable tool for precision controls and dynamic programming of cells and particles. However, conventional acoustic manipulation approaches lack the finesse necessary to form intricate, configurable, continuous, and 3D patterning of particles. Here, this study reports acoustography by Beam Engineering and Acoustic Control Node (BEACON), which delivers intricate, configurable patterns by guiding particles along custom paths with independent phase modulation. Leveraging analytical methods of orbital angular momentum beam via iterative Wirtinger hologram algorithm, this study accomplish acoustography by facilitating orbital angular momentum traps, enabling continuous 2D and 3D acoustic manipulation of microparticles in any desired geometry, with phase modulation independent of intensity. Utilizing on‐chip acoustography, the BEACON platform markedly increases the space‐bandwidth product to 31 000 while attaining an enhanced resolution with a pixel size of ≈25 µm, surpassing the typical resolution of over 200 µm in previous holographic particle manipulation methods. The capabilities of BEACON are demonstrated in creating intricate triple helical tracing structures using microdroplets (20 µm in diameter) and those carrying DNA to validate the effectiveness of the acoustography and phase control methods. This study offers new particle manipulation opportunities, paving the way for next‐generation biomedical systems and the future of contact‐free precision manufacturing.

## Graphical abstract

This study introduces BEACON, a compact, integrated acoustography platform that employs beam engineering and acoustic control nodes. Leveraging analytical methods of orbital angular momentum beam via the iterative Wirtinger hologram algorithm, BEACON achieves intricate, configurable 2D and 3D acoustic manipulation of microparticles in any desired geometry with a resolution of 25 µm and phase modulation independent of intensity.

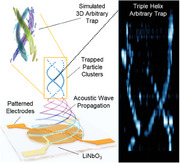

## 1 Introduction

In recent years, substantial advancements have been made possible by developing contactless tweezers,^[^ ^1^ ^]^ notably in optical^[^ ^2^, ^3^, ^4^, ^5^ ^]^ and magnetic methodologies,^[^ ^6^, ^7^, ^8^ ^]^ a progression acknowledged by Nobel Prizes in Physics in 2018. However, these techniques have encountered significant limitations, including potential photothermal or photochemical damage in cells^[^ ^9^ ^]^ and the prerequisite of pre‐tagging particles with magnetic compounds. In contrast, acoustic tweezers have gained prominence due to their label‐free nature, lower driving power, and minimal impact on cellular integrity, with power intensity ≈10 000 000 times smaller than optical tweezers.^[^ ^10^ ^]^ Acoustic tweezers are poised to transform sectors such as label‐free sensing,^[^ ^11^, ^12^ ^]^ non‐contact biocompatible particle manipulation,^[^ ^13^, ^14^, ^15^, ^16^, ^17^, ^18^, ^19^, ^20^, ^21^, ^22^, ^23^, ^24^, ^25^ ^]^ and colloid assembly,^[^ ^10^, ^26^, ^27^, ^28^, ^29^ ^]^ holding significant promise in advancing biomedical research, drug delivery, diagnostics, and therapeutics.^[^ ^30^, ^31^, ^32^ ^]^

Acoustic holography has emerged as a promising approach for achieving acoustic tweezers, notably for its ability to efficiently form patterns of microscale materials into configurable shapes in a single shot, coupled with its high level of biocompatibility.^[^ ^33^, ^34^ ^]^ Some acoustic holography approaches have utilized acoustic kinoforms to focus emission from single‐element transducers into holographic patterns at a distance.^[^ ^33^, ^34^, ^35^, ^36^ ^]^ Others have used acoustic metamaterials, harnessing subwavelength structures to control the acoustic wavefront.^[^ ^37^, ^38^, ^39^, ^40^, ^41^, ^42^, ^43^, ^44^ ^]^ Additional techniques leveraged patterned piezoelectric substrates, tailoring electrodes to modulate the acoustic field upon emission.^[^ ^13^, ^45^, ^46^, ^47^, ^48^ ^]^

The evolution of acoustic holography techniques illustrates the physical limitations of fabricating acoustic manipulation structures. Early methods employing 3D‐printed phase plates or acoustic metamaterials were constrained by the resolution limitations of 3D printing technologies (typically >100 µm) and complexities in the fabrication process such as cumbersome setups, requiring large water tanks and intricate positioning systems.^[^ ^34^ ^]^ Subsequently, kinoform‐based methods, while advancing field shaping capabilities, encountered challenges when attempting to achieve high fabrication resolution and were frequently limited to lower frequency operations (<10 MHz) due to manufacturing constraints.^[^ ^35^ ^]^ Finally, acoustic metasurfaces often require the fabrication of intricate subwavelength structures, making it challenging to produce high‐frequency systems (>10 kHz) with uniform performance across large areas.^[^ ^41^ ^]^

Although innovative, the current approaches in acoustic holography are constrained by low space‐bandwidth products due to the physical limitations in fabricating acoustic manipulation structures. These limitations adversely affect the resolution of holographic patterns. Most acoustic holography techniques depend on a surface that confines the acoustic waves using a local minimum of capillary energy, creating 2D holographic patterns with continuous tracing. However, this approach is significantly limited as it hampers the generation of stable, complex 3D tracings. The techniques cannot form continuous patterns beneath the confinement interface, making them unsuitable for intricate 3D applications.^[^ ^35^ ^]^ Moreover, existing 3D holograms suffer from the absence of a node defined by an analytical expression. This absence results in diminished fidelity due to the presence of a high‐intensity background field consisting of anti‐nodes.^[^ ^34^ ^]^

Furthermore, the process of field sculpting in previous methods predominantly relied on direct iterative numerical solutions (whereas our approach employs Fresnel‐derived analytical frameworks before proceeding to iterative refinement, providing a more precise initial condition for optimization). This reliance presents a significant challenge in generating a continuous Orbital Angular Momentum (OAM) beam, which, due to its helical phase front and orbital angular momentum, enables improved trapping stability and more precise control and manipulation of particles.^[^ ^51^ ^]^ For example, algorithms employing random phase initializations to compute holograms yield lower fidelity and precision outputs. Therefore, significant challenges remain in developing high‐resolution, configurable 3D acoustic field structuring using compact, integrated platforms. Overcoming these challenges is essential for advancing the field and expanding the potential applications of acoustic holography.

**Figure 1:**
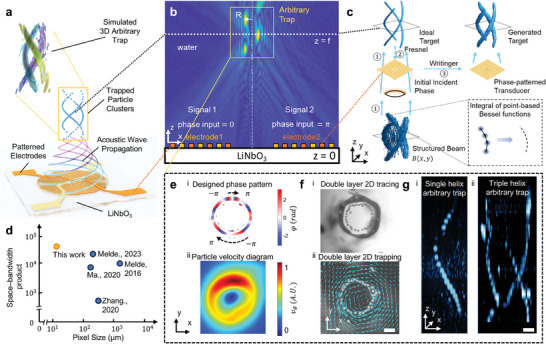
Overview of the BEACON platform. a) 3D Schematic of the BEACON platform. b) Schematic of the x‐z intersection in Figure 1a with *y*  =  0, showing the wave propagation and formation of the configurable acoustic trap. c) Demonstration of the three‐step formation of IDT with a specific target curve. Each step corresponds to an arrow in the figure, with some calculations spanning two arrows to complete one step. The yellow dashed line represents the IDT plane with *z*  =  0. d) Comparison of BEACON's accuracy with previous publications on acoustic hologram‐based manipulation. e–g) Demonstration of the key features of BEACON, including (e) configurable phase and velocity control along the curve, (f) trapping of multiple types of microparticles, and (g) particle tracing within a 3D shape. Scale bars: 50 µm.

In this work, we present acoustography by Beam Engineering & Acoustic Control Node (BEACON). This approach offers a simple yet versatile system capable of generating configurable 2D and 3D acoustic holograms with refined precision. The BEACON device consists of an analytically designed interdigital transducer (IDT) patterned on a lithium niobate substrate as a pair of electrodes (**Figure** **1**a). This enables high‐precision fabrication of complex, continuous acoustic patterns in a compact, on‐chip format using nanoscale lithography (resolution <1 µm). Utilizing IDTs eliminates the need for bulky external components and substantially reduces overall system size and complexity.

When the electrodes are actuated with radio‐frequency signals with the same waveform and a phase shift of π, acoustic vibrations with complex amplitudes will be generated from the IDT plane (*z* = 0), propagate through the liquid environment as leaky Rayleigh waves in *+z* direction, and eventually be focused near the *z*  =  *f* plane to form a 2D or 3D acoustography with a predesigned configurable shape (Figure 1b). While the initial waves generated on the substrate are surface acoustic waves (SAWs), the specific type of SAW is not critical to our method's operation. The acoustography pattern is primarily formed by leaky waves propagating directly from the electrodes into the liquid environment. These leaky waves dominate the acoustic field near the focusing plane where the pattern forms, largely unaffected by waves propagating on the substrate surface. This mechanism ensures that the acoustic phase velocity differences for different directions on the substrate have minimal impact on both our holograph formation and IDT design, contributing to the robustness and precision of our acoustography patterns. By employing the integral of point‐based Bessel beam foci to analytically express the node shapes along predetermined trajectories (Figure 1c), our approach utilizes the OAM beam to construct traps with a pixel size of less than 25 µm for particle manipulation, surpassing the typical resolution of preceding techniques (pixel size: typically > 200 µm);^[^ ^34^, ^35^ ^]^ moreover, BEACON leverages phase modulation independent of shaping the intensity distribution (i.e., defining the initial spatial patterns of acoustic intensity) to accomplish particle patterning or tracing without affecting the overall acoustic intensity (Figure 1e,i). With this approach, BEACON can realize diverse applications, including trapping particles sorted by different traps (Figure 1f,ii), tracing particle movements within complex 3D structures (Figure 1g), and achieving configurable velocity control over particles (Figure 1e,ii).

As shown in Figure 1c, our approach utilizes the integral of point‐based Bessel beam foci to analytically express the node shapes along predetermined trajectories. This technique improves spatial resolution in controlling the defocusing distance, a critical factor in constructing adaptable 3D holograms. Moreover, it allows for the independent modulation of the hologram's phase without affecting intensity, a feature not present in earlier methods.^[^ ^33^, ^34^, ^35^, ^36^ ^]^ Our strategy for hologram calculation employs Fresnel back‐propagation, significantly enhancing the stability and fidelity of both 2D and 3D acoustic OAM traps. In the field of on‐chip acoustography, the BEACON platform introduces a substantial reduction in pixel size, which is evident in the significantly enhanced space‐bandwidth products (> 31 000, see Note S1, Supporting Information for more details) and fabrication precision (Figure 1d).

The advancements delineated in this study offer enhanced flexibility and control when crafting intricate 2D or 3D patterns and overcoming previous limitations in 3D patterning capability, node fidelity, and pixel size.^[^ ^13^, ^34^, ^35^ ^]^ This advancement has the potential to unlock new opportunities in various applications, including biomedicine, material handling, and advanced sensing technologies.^[^ ^9^, ^24^, ^48^, ^49^ ^]^

## 2 Results

### 2.1 The Analytical Modeling Underpinning the BEACON Platform

In the BEACON platform, the 3D acoustic wave field formed by the IDT electrodes is vital for generating the force for particle trapping and defining the shape of the trapped particles. Acoustography, as introduced in this study, is defined as the complex 3D wave field sculptures at the focusing region near the target plane, *z*  =  *f*. This term encompasses both the generation of patterns (akin to traditional holography) and the unique capability for 3D manipulation, distinguishing our approach from existing methodologies.

Based on a three‐step analytical process, we created precise acoustic antinode patterns, as illustrated in Figure 1c. Our streamlined process is as follows:

1. Defining the target acoustography: Initially, we define the target acoustography using our primary function, *B*(*x_in_*,*y_in_*), which incorporates an integral of terms with the form of point‐based Bessel functions along the designed curve, following the definition of previous work in the field of optics.^[^ ^50^ ^]^ This step enables the creation of acoustography with an analytical expression, which is not yet achieved by existing acoustic holographic patterning methods. Despite not having a physical lens in our setup, we simulate its effect with the transformation $L\{B(x_{in},y_{in})\}$ to accurately determine the acoustography at the target plane (*B*_0_(*x_out_*,*y_out_*), also referred to as the “Ideal Target” in Figure 1c).
2. Determining the phase diagram via Fresnel transformation: Next, we establish the phase diagram at the plane found immediately after the lens (i.e., *z*  =  0), as depicted by the “Initial Incident phase” plot in Figure 1c. The focusing distance, denoted as *f*, mirrors a simulated optical system to ensure coherent back‐propagation of the complex amplitude over a distance of “*f*” to the plane right after the lens (marked as orange in Figure 1c). This step yields a phase diagram on the *x*–*y* plane, similar to a beam traversing through a lens in an optical system. This diagram serves as the initial phase diagram for our IDT pattern before refinement.
3. Phase diagram refinement using the Wirtinger hologram algorithm: Lastly, the 3D surface plot in Figure 1c (i.e., the “Generated target”) showcases the acoustic intensity distribution, derived from the refined phase diagram (i.e., the “Phase‐patterned transducer”) using the iterative Wirtinger hologram algorithm (see Experimental Section). Utilizing the Wirtinger calculus, this algorithm treats a complex‐valued function, such as the acoustic pressure field, as a bivariate function of the complex variable and its conjugate, allowing for the calculation of gradients and the formulation of optimization problems. With the phase diagram as an input, we can generate the acoustic pressure and the corresponding high acoustic intensity profile at the target plane. The algorithm iteratively optimizes the phase diagram by minimizing the error between the calculated and target patterns through gradient descent via back‐and‐forth propagation in step 2, until it converges on the optimal solution. This algorithm mitigates information loss from finite apertures during iterative propagations and enhances the representation of the desired 2D or 3D acoustography while adhering to plane constraints.

In the BEACON device, the electrodes patterned on a piezoelectric substrate generate acoustic waves upon receiving electric signals. The IDT features a dual‐electrode design. Each electrode is stimulated by a unique signal, maintaining a phase difference of π to ensure consistent referencing. To craft a coherent acoustography, we extract two isophase patterns for each electrode based on the refined phase diagram (i.e., the “Phase‐patterned transducer”), represented in Figure 1c, obtained from the Wirtinger hologram algorithm. This process requires thresholding for the generation of each electrode, considering elements such as electrode spacing that influence the acoustic field (see Note S2, Supporting Information).

### 2.2 Formation of 2D Acoustography with Configurable Shapes via BEACON

Our method crafts configurable tracings by integrating OAM beam spots while retaining innate OAM properties. In this work, we devised an Ideal Target, *B*_0_(*x*,*y*), which enables the generation of acoustic beams with uniform intensity and an adaptable phase distribution along a predetermined shape function, ξ(*t*)  = (*x_T_* (*t*),*y_T_* (*t*),*z_T_* (*t*)) :^[^ ^50^, ^51^ ^]^

(1) $$\begin{array}{ccc} B_{0}\;\left(x_{out},y_{out}\right) & = & \;L\left\{B\left(x_{in},y_{in}\right)\right\}\;\\ & = & \;L\left\{\frac{1}{\int_{0}^{T}C\left(t\right)dt}\int_{0}^{T}\exp\left(\frac{4\pi^{2}r^{2}i}{\lambda^{2}f^{2}m}\left[y_{in}x_{T}\left(t\right)-x_{in}y_{T}\left(t\right)\right]\right.\right.\\ & & \left.\left.+i\frac{2\pi m\phi \left(t\right)}{\phi \left(T\right)}\right)\psi \left(t\right)C\left(t\right)dt\right\} \end{array}$$

with the transformation L{f(x,y)} of any function *f*(*x*, *y*) defined as

(2) $$L\;\left\{f\left(x,y\right)\right\}=\frac{1}{4\pi^{2}}\;\int \;\;\;\int f\left(x,y\right)\exp\left(2\pi i\left(ux+vy\right)\right)dxdy$$

and

(3)

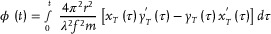

(4) $$\psi \;\left(x_{in},y_{in},t\right)=\;\exp\left(i\pi \frac{{\left[x_{in}-x_{T}\left(t\right)\right]}^{2}+{\left[y_{in}-y_{T}\left(t\right)\right]}^{2}}{\lambda f^{2}}z_{T}\left(t\right)\right)$$

(5)

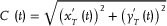

where λ is the wavelength of the acoustic wave, *f* is the designed focusing distance of the IDT, *m* is the topological charge affecting phase change along the target, *r* is a factor that controls the scale of the target, and *f*′(*x*) represents the derivative of any function *f*(*x*).

**Figure 2:**
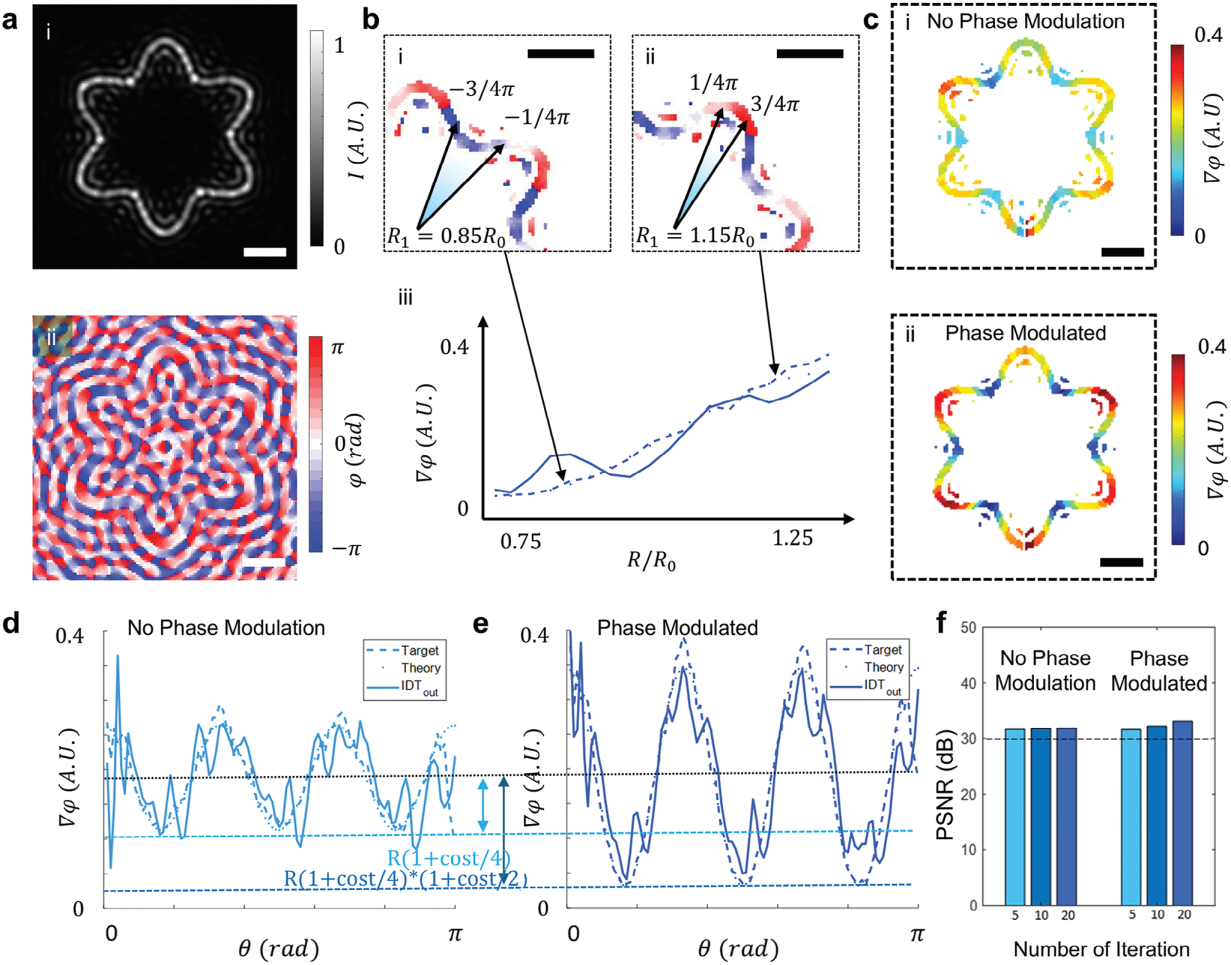
Simulating analytical configurable 2D trap and independent phase diagram modulation in acoustography. a) The intensity (i) and phase diagram (ii) depict a Michelin star contour, demonstrating the successful tuning of the phase to a configurable setting. b) Illustration of the relation of phase gradient with the distance of the point on the curve to the origin, R. The phase difference between the black arrows is π/2. c) The target phase gradient without (i) and with (ii) modulation of the phase gradient term, ϕ(*t*). The phase difference between the black arrows is π. d,e) The phase gradient distributions without (d) and with (e) modulation of the phase gradient term. f) The peak signal‐to‐noise ratio shows constant high values (> 30 dB) over several iterations of the Wirtinger hologram algorithm, confirming the efficacy of our approach in maintaining the OAM properties of the acoustography while allowing for phase modifications. The scale bars are 50 µm.

It is worth noticing that the flexible term ϕ(*t*) in Equation (3) governs the design of the phase gradient. Adjusting ϕ(*t*) allows precise alignment of the phase gradient for tracing velocity control independent of curve geometry modifications. This modification allows the particles to traverse the curve at varying speeds while maintaining structural integrity, overcoming a limitation inherent in conventional Bessel beams. For example, **Figure** **2**a,i,ii depicts intensity and phase diagrams resembling a Michelin star contour, showcasing the successful generation of the phase and intensity in a configurable setting. For enhanced clarity, the following phase diagrams selectively exhibit only the phases within high‐intensity areas, filtering out regions where intensity falls below 50% of the maximum. With the unmodified form of the phase term, ϕ(*t*), as shown in the equation above, the phase gradient along the curve, ∇φ, is proportional to the distance of each spot of high‐intensity area to the origin in *x*–*y* plane, R (Figure 2b). In Figure 2b,iii, the phase gradient of the target curve (illustrated by the dotted line) aligns closely with the simulation results (depicted as a line plot), particularly as the value of R increases along the curve. The black arrows effectively correlate the actual localized phase pattern (shown in Figure 2b,i,ii) with two distinct points on the curve, each characterized by a different value of R. Furthermore, the ϕ(*t*)/ϕ(*T*) term ensures phase alignment at both the starting and ending points of the beam (*t*  =  0 and *t*  =  *T*), preventing potential isophase disruptions that form the acoustography and maintaining a unified and robust acoustography profile.^[^ ^51^ ^]^

Through the manipulation of ϕ(*t*), we can engineer a phase gradient for specific applications while maintaining an accurate intensity distribution as designed. Figure 2c,i,ii, which depict the generated phase with and without the modulation of ϕ(*t*), validate this result by demonstrating a change in the phase diagram. Figure 2d,e illustrates the phase gradient distributions for the two patterns, respectively, to realize tracing velocity control. The difference in the phase gradient pattern and the alignment of the target and simulation for both results demonstrate the efficacy of our approach for configurable phase modulation. The peak signal‐to‐noise ratio of the intensity pattern shows a constant level of > 30 dB over 20 iterations of the Wirtinger hologram algorithm (Figure 2f), which is generally considered a satisfactory level for high‐fidelity images in previous works.^[^ ^52^ ^]^ This result confirms the efficacy of our approach in preserving the OAM properties of the acoustography while facilitating phase modifications.

### 2.3 Generation of 3D Acoustography via BEACON

**Figure 3:**
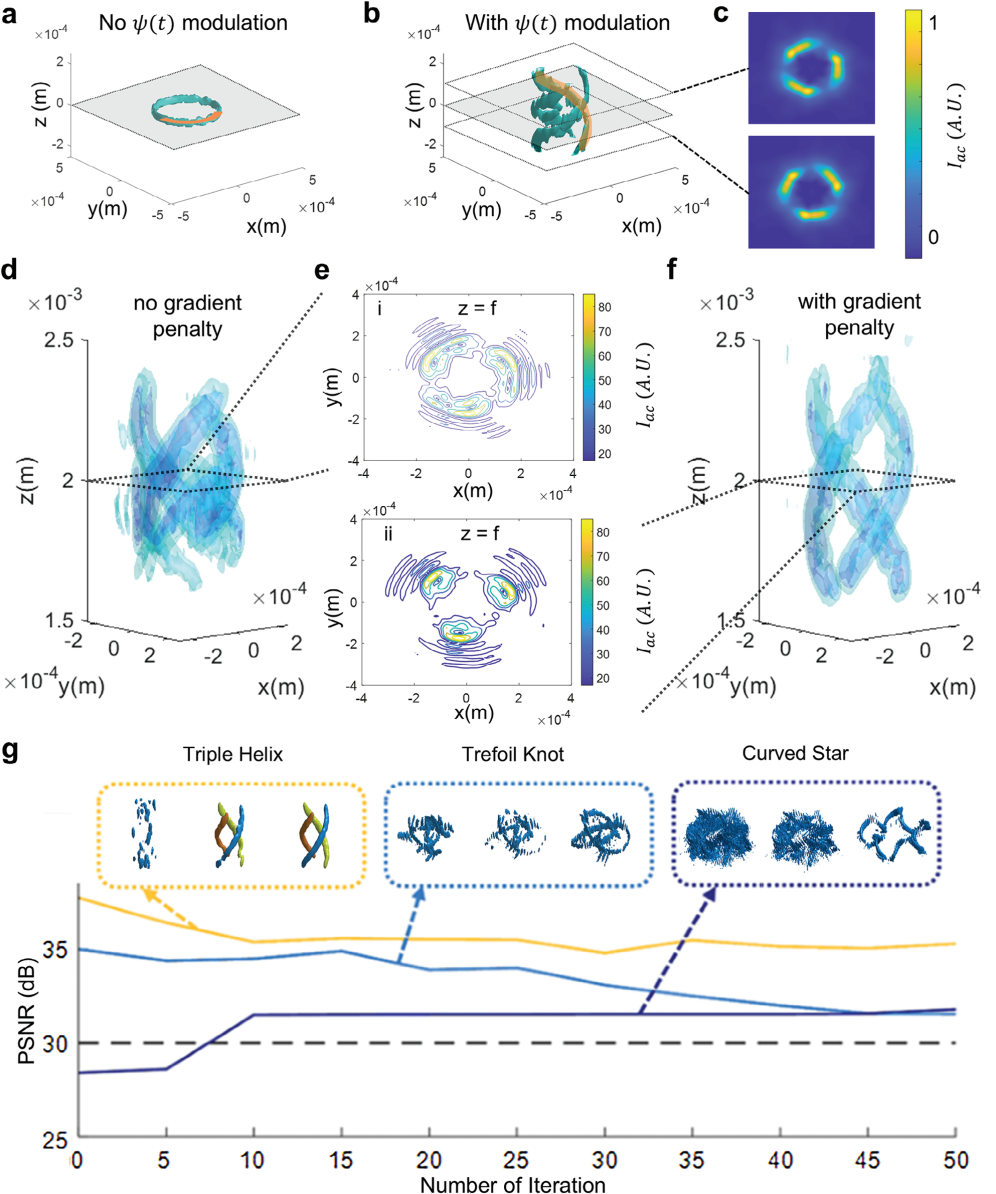
Simulation of 3D acoustography. a,b) A triple helix shape is depicted both with and without our defocusing term, illustrating the transition from a 2D node to a 3D node. The part marked with orange represents the same part of the node with and without our defocusing. c) Two different *x*–*y* intersection views below and above the *z*  =  0 plane respectively, showing the 3D structure of the target node. d,f) The penalty term of the algorithm enhances the intensity gradient of the traced contour, indicating a successful optimization process with a more condensed contour, indicative of a larger gradient. e) Intersections of the *x*–*y* plane reveal closer contours, confirming the effectiveness of the optimization. g) For different 3D nodes, the evolution of peak signal‐to‐noise ratio throughout the iterations indicates a value above 30 dB, affirming the method's efficacy in maintaining a high level of precision. The contour plot for each node is captured with 1, 10, and 50 iterations, respectively.

Following the illustration in Section 2.2, our approach enables the production of acoustography shaped in configurable 3D curves, enhancing acoustic field control beyond traditional 2D configurations. This advancement is realized by modulating the quadratic phase function, ψ(*x*, *y*, *t*), which is influenced by a predefined curve that encompasses (*x_T_* (*t*),*y_T_* (*t*)) and a variable defocusing distance, *z_T_* (*t*). This modulation facilitates the assembly of new beam terms, enabling the creation of complex 3D structures without altering the acoustography's focal properties. **Figure** **3** explains the efficacy of our methodology. Figure 3a depicts a configuration reminiscent of Figure 2a with triple helix‐shaped structural elements, yet it introduces a phase modulation term that signifies the transition from 2D to 3D forms. As shown in the orange marked segments in Figure 3a,b, with the addition of a specific ψ(*x*, *y*, *t*), part of the circular acoustography is projected into a helix shape as a part of the corresponding 3D acoustography. The transformation from the initial structure underscores our ability to generate 3D configurable acoustography without compromising fidelity. In Figure 3c, the intersection presented on two different x–y planes, with i) *z*  =  *f* − 100 µm and ii) *z*  =  *f* + 100 µm, respectively, provides a detailed cross‐sectional view of the 3D configuration.

As the acoustic radiation force on a particle is defined by the spatial gradient of Gor'kov potential (Note S3, Supporting Information), a highly localized intensity on the acoustography is vital for efficient particle trapping.^[^ ^53^, ^54^ ^]^ The resulting acoustography intersection shown in Figure 3e,ii ensures the generation of sufficient force for particle manipulation and elucidates the level of precision achieved through our approach. The foundation of this methodology is an original cost function in the Wirtinger hologram algorithm that facilitates the independent modulation of the intensity gradient. Combining the cost function with a penalty of the 3D target based on the intensity gradient in the z direction, this approach overcomes constraints associated with previous methods,^[^ ^34^ ^]^ as evidenced by the difference of the multilayer intensity contours in Figure 3d,f. Integrating the penalty term refines the trace contour's intensity gradient, resulting in more precise and defined patterns. Further scrutiny of Figure 3e reveals intersections on the *x–y* plane, emphasizing the proximity of the contours and attesting to the success of the optimization process. The five different contours in each image show the boundaries of the absolute acoustic intensities in the intersection, with the part of light yellow indicating the maximum region. Compared with Figure 3e,i, the enhanced gradient in Figure 3e,ii validates the proficiency of our optimization, manifesting in a well confined contour. With the addition of a modulation term in the cost function, the peak signal‐to‐noise ratio might have a slight decrease due to the intensity gradient‐based penalty within the iteration process but will remain consistently at a high level of > 30 dB. The trajectory depicted in Figure 3g illustrates the peak signal‐to‐noise ratio and relative intensity contour of three acoustographys with different shapes (i.e., triple helix, trefoil knot, and curved star) across 50 iterations. As the iteration number increases, the intensity contour of each of the shapes converges from scattered points to a better‐confined 3D trace. This result affirms the precision of our method to consistently improve the shape of configurable 3D acoustographys while maintaining a high‐intensity gradient.

### 2.4 Demonstration of 2D Particle Patterning and Configurable Phase Control via BEACON

The BEACON platform utilizes patterned gold electrodes on a LiNbO_3_ substrate to generate an acoustic field that traps microparticles at either pressure nodes or antinodes within a sealed polydimethylsiloxane (PDMS) chamber. The chamber height is set to 300 µm above the focusing distance to position the trapping plane at the focal point, while its configuration (open or closed) and positioning (above or below the substrate) are tailored to enhance trapping efficiency for the target particle type suspended in the fluid medium. (For more details on the system setup, see Experimental Section.)

**Figure 4:**
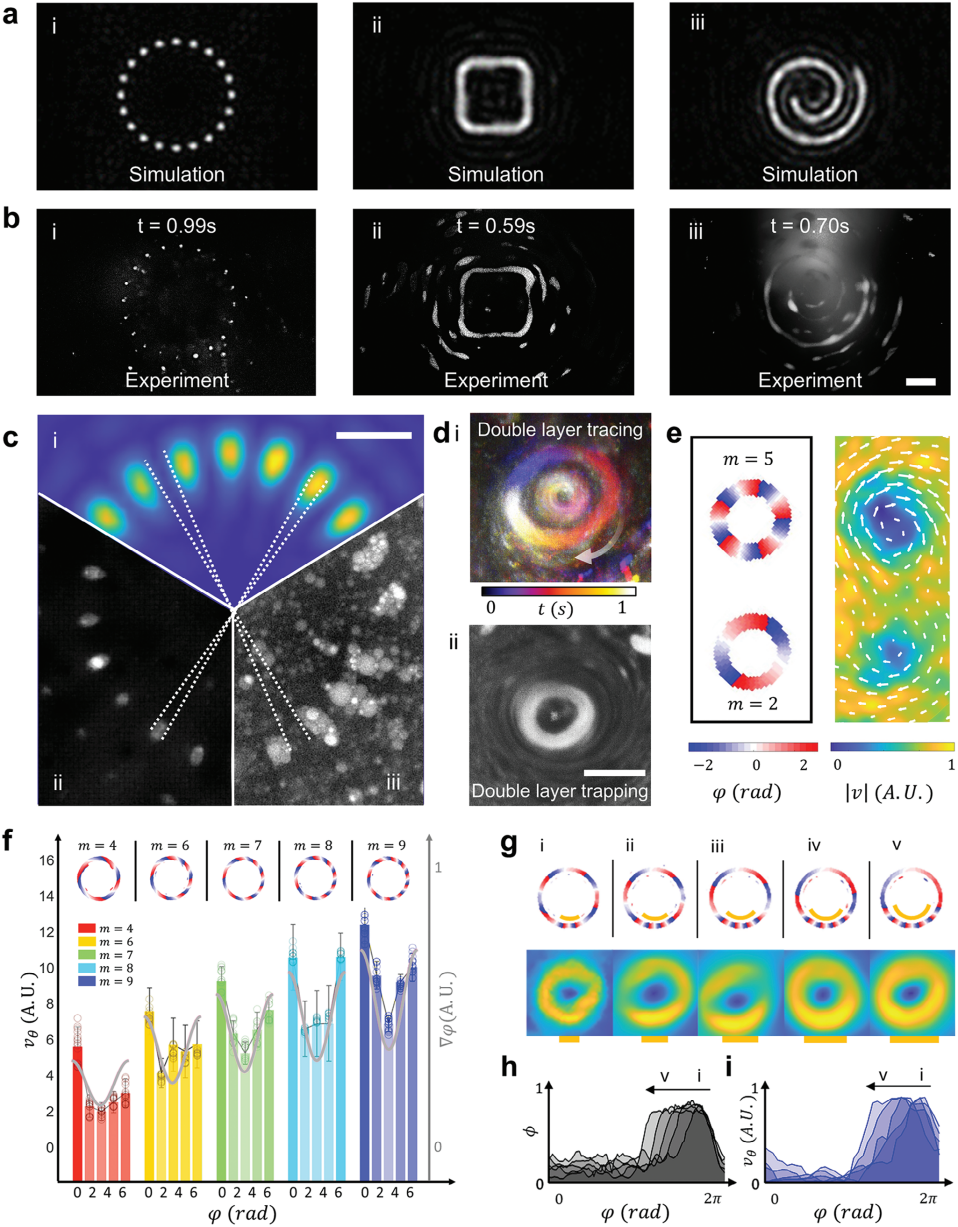
Experimental validation of 2D configurable tracing and phase diagram modulation. a,b) A comparative analysis showcasing the synchronization between (a) simulated intensity diagrams and (b) actual patterns formed by trapped heptane microdroplets in a DI water environment, highlighting the rapid patterning capabilities of our method. c) Demonstration of BEACON's resolution capabilities. (i) Simulated acoustography with 20 dots in a circular arrangement. (ii) Experimental result using a low concentration of heptane microdroplets. (iii) Experimental result using a high concentration of heptane microdroplets. The average distance between adjacent high‐intensity regions is 24.43 ± 5.55 µm. d) Illustration of tracing and trapping of heptane and PDMS particles within one ring‐shaped acoustography. e) Modulation of particle velocities within a single IDT through varying topological charges of the ring‐shaped pattern. f) A demonstration of the congruence between speed distribution and respective topological charges, complemented by a particle image velocimetry analysis depicting the speed vectors of tracing particles, highlighting the precision and versatility of our method in manipulating particle trajectories and speeds. g) Comparison of the simulation of phase diagrams with the increasing length of the high‐intensity segment (marked yellow) and the corresponding particle image velocimetry results. h,i) A detailed investigation into the implications of introducing a segment with heightened intensity within ring‐shaped patterns in (g), substantiated by simulated normalized phase gradient distribution (h), elucidating the direct relationship between the length of the high‐intensity segment and particle velocity (i). Scale bars: 80 µm.

With preliminary simulations, our experimental results revealed the performance of our approach to generate 2D patterns with acoustography. **Figure** **4**a,b specifically delineate three configurable patterns: a ring of dots, a square, and a spiral, with the bright part indicating the 50% high‐intensity contour for Figure 4a and the concentrated fluorescent heptane microdroplets for Figure 4b, respectively. Notably, in Figure 4b,i, the dot formation within the pattern, in reference to the scale bar, demonstrates a high spatial‐bandwidth product (Note S1, Supporting Information). With the acoustics turned on, the heptane microdroplets with a negative acoustic contrast factor are brought to the acoustography by streaming. This patterning at the *z*  =  *f* plane occurs due to the acoustic radiation force, with the duration of the pattern formation process, denoted as t, being less than 1 s (Figure S1d and Movie S1, Supporting Information). For acoustography shapes with continuous traces, the trapped particles will keep moving along the trace of the acoustic antinode with a velocity proportional to the magnitude of the designed phase gradient. For acoustography with open shapes, such as the spiral, trapped particles will eventually leave the acoustography at the endpoint of the antinode; if the acoustography pattern is enclosed, such as the square, trapped particles will keep orbiting until the acoustic signal is turned off. The alignment between the simulated intensity diagrams and the actual patterns formed by trapped heptane microdroplets proves the fidelity and the rapidness of patterning.

To illustrate the precision and resolution of our acoustography system, we conducted experiments using heptane microdroplet dispersions at different concentrations (Figure 4c). The target acoustography, a circular arrangement of dots (diameter: 320 µm), is shown in the simulated acoustic intensity field (Figure 4c,i). With a low concentration of droplets, we observed a clear circular arrangement of distinct dots, accurately representing the positions of maximum acoustic intensity (Figure 4c,ii). Using a higher concentration, heptane microdroplets accumulated in all high‐intensity areas, with clearly defined low‐intensity regions between adjacent dots (Figure 4c,iii), demonstrating the accuracy of our acoustography in maintaining both high and low‐intensity area dimensions.

A further testament to the method‘s versatility is provided in Figure 4d, wherein particles of varied acoustic contrast factors are trapped in different parts of the acoustography, from PDMS particles to their negative counterparts, heptane of contrast factor −1.07 (for the preparation of PDMS particles, see Material and Methods; for the estimation of acoustic contrast factors, see Note S3, Supporting Information). The time‐lapsed image with a rainbow color shows the tracing of heptane microdroplets. The PDMS particle is trapped in the center, while the heptane microdroplets circulate following a ring trace around it. Movie S2 (Supporting Information) also demonstrates the trapping of particles with different acoustic contrast factors. While the heptane microdroplets circulate following a ring trace, the polystyrene particles of contrast factor 0.49 circulate following another inner ring trace near the pressure node.

After verifying the 2D patterning of different acoustographys, we test the performance of our method to control phase distribution by generating an acoustography with the shape of two adjacent circles with different topological charges and measuring the velocity via a particle tracing experiment (Figure 4e). With the same ring‐shaped phase diagram of m = 2 and 5, the particle image velocimetry results show a distinctive difference in mean particle velocity as designed. In Figure 4f, we utilize a series of ring‐shaped phase diagrams containing points with enhanced velocities at different topological charges. The phase gradient of each contour has the same trend along the trace but is scaled by the topological charge value, m = 4, 6, 7, 8, and 9, as shown in the phase diagrams in Figure 4f. The five groups of bar graph show the particle velocity measured at four evenly distributed points (φ  =  0,  π/2,  π, and 3π/2) along each of the ring traces, with the error bar as the speed variance for each group of measurement. As expected, based on the designs of the ring‐shaped contours, the change of particle velocity shares the same trend among all groups (to have sides with high velocity and center with low velocity), and the average velocity magnitude of each group is proportional to the topological charge of the corresponding contour. The experimental result of the particle velocity trend matches well with the designed phase gradient (as the dotted line on each group), showcasing our method‘s precision in controlling the particle velocity. Furthermore, Figure 4g‐i delves into the expanded capabilities of our phase control technique. In Figure 4g,i‐v, five ring‐shaped acoustographys with an increasing segment (marked by orange lines) with enhanced phase gradient are designed. This approach establishes a clear connection between the high phase gradient segment's length and the resultant particle velocity, as shown in the particle image velocimetry results in Figure 4g,i‐v.

The results from the five acoustographys are plotted together as (i–v) in Figure 4h,i. The x‐axis in both figures represents the relative angular position on the ring curve, ranging from 0 to 2π. In Figure 4h, the y‐axis shows the designed phase gradient, while in Figure 4i, it displays the normalized particle velocity along the curve, calculated based on particle image velocimetry results. An arrow indicator in both figures points to a series of curves labeled (i–v), with the peaks of these curves shifting from right to left. These curves correspond to the five ring‐shaped acoustographys with increasing lengths of high phase gradient segments, as illustrated in Figure 4g. The shape of the curves in both figures shows similar patterns, indicating a direct influence of the designed phase gradient (Figure 4h) on the particle velocity (Figure 4i). As we progress from curve (i–v), we observe an expansion of the high‐value segments in both the phase gradient and particle velocity. This expansion indicates that a larger portion of the ring experiences increased phase gradient and, correspondingly, higher particle speed. The overlapping colors in the graphs represent the consistency of this relationship across different designs. The small inset figures correspond to the different acoustography designs shown in Figure 4g, providing a visual reference for each curve.

These trends demonstrate that by manipulating the phase gradient along the ring, we can precisely control the particle velocity at different angular positions. The correlation between the angular position and particle velocity follows the designed phase gradient, allowing for tailored particle movement along the acoustography pattern. This relationship holds true across the entire 0 to 2π range, showcasing the fine control our method offers over particle dynamics in circular acoustography patterns.

### 2.5 Demonstration of 3D Particle Tracing via BEACON

**Figure 5:**
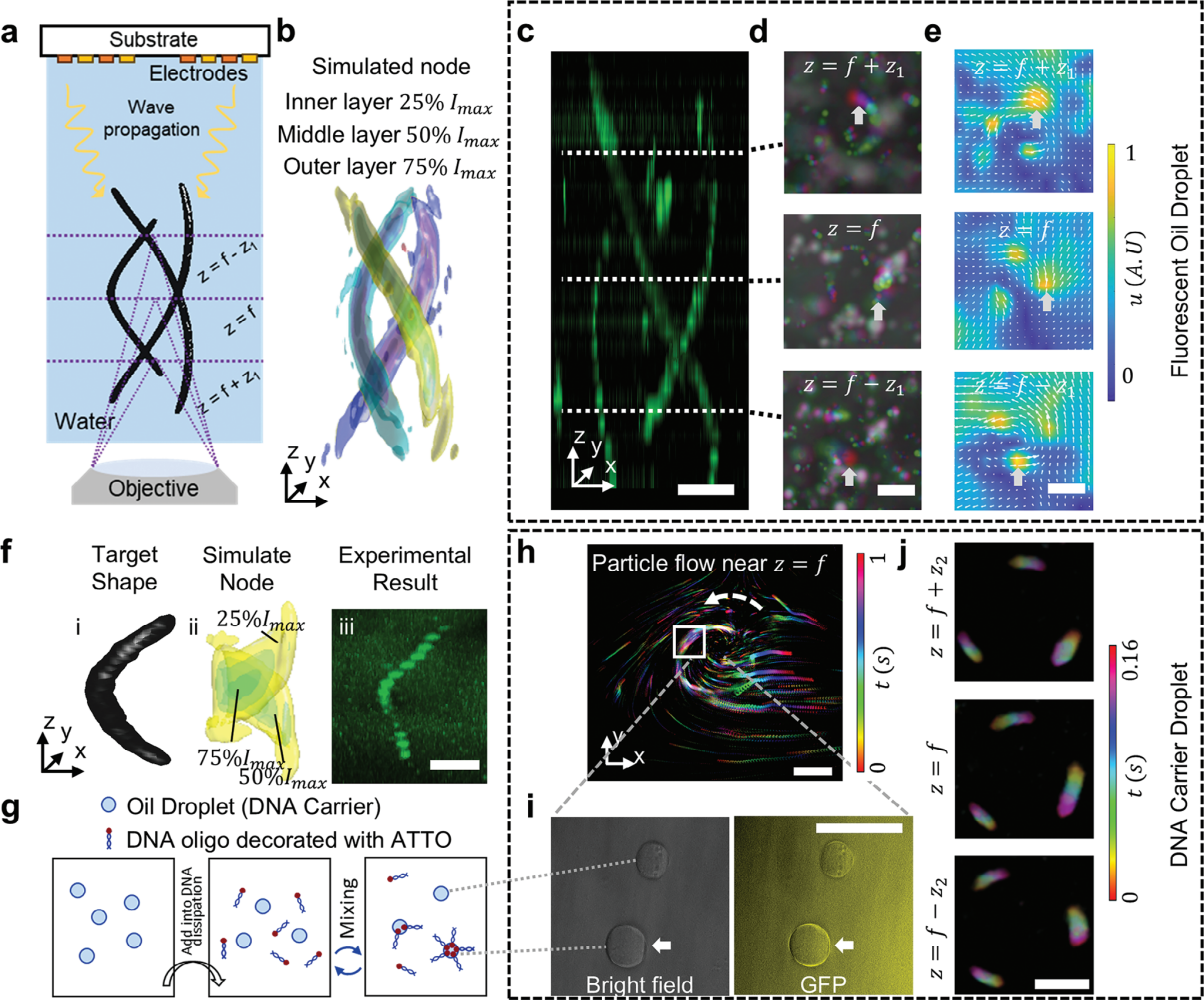
Experimental realization of 3D triple helix tracing and rapid DNA carrier manipulation via BEACON. a) Schematic for the setup for triple helix structure imaging. b) Illustration of simulated intensity distributions within a triple helix bundle configuration, showcasing the potential of the Wirtinger hologram algorithm in enhancing the localization of regions with elevated intensity. c) Confocal microscopy representation of particles tracing along a 3‐D helical structure. d) Time‐lapsed photos with *x*–*y* intersection views at different z positions of Figure 5b. e) corresponding particle image velocimetry result of (d). f) (i) Target shape, (ii) simulated intensity distributions within a singular‐helix configuration, and (iii) confocal imaging result. g) Schematic of DNA carrier droplet formulation, illustrating the encapsulation process of DNA molecules onto the surface of oil microdroplets labeling them with fluorescent for subsequent manipulation. h) Time‐lapsed visualization of DNA carriers maneuvering along predefined paths. Each part of the trace is generated by time stacked image of particle movement of DNA carrier particles, as shown in Figure 5d. i) Depiction of the creation of distinctive DNA carriers capable of capturing dispersed DNA molecules. The white arrows show the particles carried with fluorescent DNA. j) Three different time‐lapsed *x*–*y* intersection views at different z positions. Scale bars: 100 µm.

In this section, we further validate our method of crafting complex 3‐D structures. **Figure** **5**a illustrates the schematic of our experimental setup: the device is inverted, with the patterned side facing downward toward the chamber that is affixed beneath the substrate. Observations are made using an inverted microscope positioned below the chamber, focusing on three distinct planes (*z*  =  *f* − *z*_1_,*f*, *f* + *z*_1_, respectively, with *z*_1_ =  100 µm and *f*  =  2 mm) to capture videos of the acoustic manipulation process. The idealized acoustography, depicted in black, represents the triple helix structure we aim to emulate.

Figure 5b offers a simulated representation of the targeted 3D configuration, particularly a triple helix. The simulation delineates three layers—inner, middle, and outer—to represent the intensity contour with 25%, 50%, and 75% of the maximum intensity of the node, respectively. Integrating a term into the cost function is the key to achieving such an intensity gradient. By doing so, we have been able to counteract the repelling force,^[^ ^53^ ^]^ paving the way for polymerizing sophisticated 3D patterned particles with high resolution. Figure 5c serves as a testament to our method‘s applicability. We utilized confocal microscopy to transform our envisioned 3D structure into a tangible reality. The scanning across different *z*‐planes renders a comprehensive 3D representation. Similar to the case with a 2D setup, the particles with negative acoustic contrast factors will be attracted to the node when the acoustic power is turned on. The particles trapped within the 3D node will move slowly, forming a train inside the acoustography, where they follow each other tightly. This particle movement translates to the tracing of particles within traps, with the timelapse images of different *x*–*y* intersections further demonstrating the shape of the 3D tracing (Figure 5d). The trains of microdroplets localizing within our triple helix trap offer a gateway to emulate complex micro‐structures inherent in biological entities, like collagen. Such advancements could reshape fields like tissue engineering and regenerative medicine.

Figure 5e presents the particle image velocimetry analysis results, offering a vivid depiction of particle dynamics as observed in Figure 5c. The color map within each plane indicates the velocity magnitude of the particles, while the superimposed vectors clearly delineate their directional movement. This visual analysis confirms that the particles are not only moving along the predefined trace but are also doing so at consistent velocities, as evidenced by the uniformity of the color gradients. The vectors are tightly aligned with the trace paths, underscoring the precision of our trapping and guidance system. The sequential timelapse images across various *x*–*y* planes showcase the continuity and shape of the 3D tracing, capturing the orchestrated movement of particle trains along the intricate paths designed by our BEACON platform.

While Figure 5f,ii offers a simulated representation of the targeted 3D configuration with a shape of a singular helix, verifying the ideal shape as Figure 5f,i, we spotlight the importance of the phase distribution along the singular helix trace. Maintaining a balanced topological charge prevents unwanted particle stagnation and ensures a fluid, uninterrupted particle trajectory. While we could successfully generate a single or triple helix acoustography with a negative topological charge, the Fresnel propagation will generate a phase profile with a high noise level along the helix. Thus, we instead use a small and positive topological charge to ensure a consistent phase distribution along the trace (Figure S3, Supporting Information). This intricacy ensures the precision with which we can realize our designs. Figure 5f,iii presents a confocal image that serves as empirical verification of the simulation depicted in Figure 5f,ii. This image confirms the precision with which hexadecane droplets are patterned, aligning with the singular helix shape outlined in Figure 5f,i. The confocal image not only corroborates the simulated structure but also emphasizes the critical role of phase distribution in achieving the fidelity of the helical trace, ensuring that the practical application of our methodology aligns with theoretical predictions. In all the experiments depicted in Figure 5c–f, we have consistently utilized hexadecane particles dyed for visibility, each with a diameter of ≈20 µm. Building upon this foundation, we will delve into a promising application involving DNA carrier particles.

Figure 5g explores an original methodology to encapsulate distinct DNA carriers characterized by specific DNA molecules attached to their surface (see Material and Methods). The inclusion of DNA oligos, decorated with Atto molecules on one end, serves as markers, facilitating the differentiation of DNA‐rich droplets under fluorescent illumination. Initially, oil microdroplets of ≈20 µm in diameter are formed in a DNA‐free buffer and subsequently introduced into a DNA dispersion. With different polarities on each side of the chain, the DNA molecules can be attracted and captured by the aqueous‐oil interface (i.e., the surface of the oil microdroplets), forming a fluorescent DNA carrier to be used for successive manipulation. This result emphasizes the strides we have taken in integrating genetic materials with acoustic manipulations, introducing a new layer of complexity and control in our experiments.

Figure 5h provides a detailed view of our acoustic manipulation capabilities, showcasing the DNA carriers maneuvering predefined trajectories at the middle plane of a single helix trap. Lastly, we move back to the triple helix acoustography, as in Figure 5i, and pattern DNA carrier droplets with it (Movie S3, Supporting Information). Figure 5j shows three time‐lapsed images at different *z* positions with *z*  =  *f* − *z*_2_,*f*, *f* + *z*_2_, respectively, with *z*_2_ =  50 µm and *f*  =  2 mm. The gradual change of the focusing position as z increases in Figure 5j matches the simulation results shown in Figure 5b. It is not just a showcase of the method‘s functionality but a demonstration of the temporal intricacies involved in the manipulation process.

In summary, this section highlights the potential of our BEACON technique. It paves the way for polymerizing contents within the chamber, resulting in patterned DNA throughout the medium, and utilizes self‐adhesive carrier droplets to assemble patterned DNA carriers into a cohesive unit that can be seamlessly extracted from the medium. Similar to hydrogel microdroplets, these structures formed by DNA carrier droplets have the stability, biocompatibility, and tunable physical properties for potential biological applications such as tissue engineering in future works.

## 3 Discussion

The BEACON system enables the creation of configurable 2D and 3D acoustographys with independently modulated phase diagrams. This approach allows for continuous acoustic nodes along closed shapes with a minimal spacing of ≈2λ for the current setup's aperture. The alignment of both phase and phase gradient at the starting and ending points of the prescribed enclosed curve (t = 0 and t = T) ensures constant intensity along the curve, preventing breakpoints due to phase interference.

Building upon previous work in 3D particle trapping,^[^ ^53^ ^]^ our method introduces precise manipulation of particles within 3D structures with phase control independent of acoustic intensity. This approach enables on‐chip particle tracing along configurable paths in 3D, expanding on prior acoustic holography research^[^ ^16^, ^33^, ^35^ ^]^ that primarily focused on non‐Orbital Angular Momentum (OAM) beams. OAM beams, characterized by their helical phase front, offer improved trapping stability and more precise control of particles. The BEACON platform increases the space‐bandwidth product to 31 000 and achieves a resolution with a pixel size of ≈25 µm, compared to the typical resolution of over 200 µm in previous holographic particle manipulation methods. Our system's pixel size, defined as the smallest independently controllable unit of the acoustic field, is fundamentally limited by the operating frequency and the space‐bandwidth product. While recent methods have demonstrated pattern generation capabilities with sizes under 100 µm, their effective pixel sizes remain constrained by these factors. For instance, systems operating at frequencies up to 15 MHz are theoretically limited to a minimum pixel size of ≈80 µm, assuming a speed of sound of 2400 m s^−1^ in the hologram medium. Furthermore, the actual pixel size in these systems is often larger due to limitations in their space‐bandwidth products. BEACON's higher operating frequencies (22–30 MHz) and larger effective aperture enable us to achieve a smaller pixel size, allowing for more precise control of the acoustic field. The BEACON system utilizes phase modulation independent of shaping the intensity distribution to achieve high‐fidelity acoustographys, evidenced by a peak signal‐to‐noise ratio exceeding 30 dB. This represents a significant improvement in resolution and control precision compared to conventional acoustic manipulation techniques, which typically achieve a resolution of ≈200 µm.

It's worth noting that our characterization of other platforms' pixel sizes as ≈200 µm is not based on an explicit statement in their publications. This value stems from our specific approach to defining and calculating pixel size, which is the smallest independently controllable unit of the acoustic field. This definition is closely tied to the concept of space‐bandwidth product (SW), as it is correlates to the smallest number of independently controllable units of the system. Regarding the SW, we employ this metric as a comprehensive measure of acoustography performance. The SW is defined as SW = N × N = (L/d)^2^, where L represents the lateral dimension of the acoustography and d denotes the scale of the smallest distinguishable unit on the IDT. This product quantifies the information capacity of holograms, effectively characterizing the intricacy of the wavefront that can be generated. For a comprehensive explanation of our approach to defining pixel size, our method for characterizing other platforms resolution, and a detailed calculation of SW, please refer to Note S1 (Supporting Information).

Two‐phase extraction from the optimized phase diagram plays a crucial role in determining acoustography fidelity. Note S2, Figures S4 and S5 (Supporting Information) illustrate how different phase choices influence results, highlighting the need for careful balance to prevent information loss from discarded phases. The use of Fresnel‐derived continuous isophase spirals in the beam function design results in a flattened device topology, simplifying the platform.

This work primarily focuses on trapping or tracing particles with negative acoustic contrast factors. Patterning particles with positive acoustic contrast factors along a prescribed curve presents challenges, requiring an acoustic field with high background pressure but zero pressure along the curve. The high operating frequency (22–30 MHz in this work) and low‐viscosity liquid environment can create significant acoustic streaming, leading to unstable patterning. Additionally, the heating effect from high acoustic pressure in the background may not be suitable for biological samples. Addressing these challenges in future work could expand the applicability of the BEACON system to a wider range of particles and biological contexts.

Looking ahead, the BEACON system opens new possibilities in materials science and biological applications. It has the potential to enable the synthesis of bioinspired materials mimicking intricate biological formations, such as 3D biopolymers with tunable properties for biofabrication and tissue engineering. The system's ability to generate configurable phase diagrams for 2D patterns also presents opportunities for manipulating “floating” acoustographys, offering new approaches to acoustic manipulation experiments.

As research progresses, we anticipate that the applications of this system may extend beyond the laboratory, potentially finding use in various industries and technologies that require precise acoustic manipulation. The high resolution and control precision offered by BEACON could lead to advancements in fields such as drug delivery, additive manufacturing, and biomedical microsystems.

## 4 Experimental Section

### The Wirtinger Hologram Algorithm

To obtain a phase diagram that can generate a target acoustography with high fidelity, the iterative algorithm based on the Wirtinger hologram was used to generate the output phase to generate a high‐fidelity acoustography.^[^ ^52^,^55^ ^]^ The initial target node and the phase diagram were generated by the Fresnel diffraction method. Compared with other methods, such as random‐phase initialization, using the initial phase diagram as an input can improve the fidelity of the node outcome in the optimized phase diagram (Figure S7, Supporting Information).^[^ ^51^ ^]^ In the iteration process, the Fast Fourier Transform (FFT)‐based angular spectrum method was used instead to calculate the free propagation and back‐propagation of the acoustic wavefront.^[^ ^56^ ^]^ This method relies on i) the FFT of the complex phase diagram, ii) the propagation of each corresponding wave component in the frequency space to a target plane, and iii) the inverse FFT of the result.

The process of the algorithm can be described as follows:

1. Use the initial phase diagram and target node intensity as input;
2. Propagate the phase diagram to multiple planes near the target plane and calculate the cost function;
3. Find the direction of the phase vector with the steepest descent of the cost function. In particular, the mean‐squared error was used together with a factor controlling the loss of intensity gradient as the cost function;
4. Update the phase diagram based on the phase vector and step size;
5. Repeat steps 2–4 until the maximum number of iterations was reached or the change in cost function within one iteration was below the threshold.

For the iteration process, both the phase diagram in IDT plane (Φ) and the acoustic intensity in the target node plane *I*(*x*, *y*) was stored in a real matrix of 750*750 pixels. The optimization process can be described as

(6) $$\;\Phi_{\mathrm{optimized}}=\underset{\Phi }{\mathrm{minimize}}\;Err\left(H\left(\Phi ,f\right),I_{target}\right)+\gamma ∥\nabla \Phi {∥}^{2}\;$$

where H(Φ,f) is the acoustic intensity of Φ after propagating to the target node plane at *z = f*, γ is a weighing factor of the gradient, and ‖*‖ stands for pixelwise sum‐up. In this work, the *l_2_* penalty was used as the error function, $Err\;\left(I_{1},I_{2}\right)=\frac{1}{2}\;∥{\left(I_{1}-I_{2}\right)}^{2}∥$.

### General Experimental Setup for the BEACON Platform

With the definition of Gor'kov potential, particles with a size much smaller than the wavelength will be attracted to either acoustic pressure nodes or antinodes, depending on the sign of its acoustic contrast factor. Thus, the formation of high‐intensity acoustography functions as antinodes, effectively creating tracing traps for particles exhibiting a negative contrast factor (Note S3, Supporting Information).^[^ ^57^,^58^ ^]^ Conversely, when the acoustography forms a small, enclosed pattern, spanning a diameter of several wavelengths, the center acts as a trap for particles with positive contrast factors. For most of our experiments, heptane microdroplets suspended in water were utilized to represent negative contrast factors, while PDMS particles were chosen to represent positive contrast factors. By tuning the formulation of the curing agent, the mechanical properties can be adjusted significantly,^[^ ^59^ ^]^ making the possible acoustic contrast factor of the PDMS microparticles range from negative to slightly positive. To create PDMS microparticles with a positive acoustic contrast factor, it use a high wt% of the curing agent (1:5) and a long baking time to increase the stiffness of the PDMS microparticles while keeping its density slightly larger than the density of water.

As it was challenging to directly measure the mechanical properties of the PDMS microparticles generated in this work, experimental tests were performed with 2D patterning in standing acoustic waves using a mixture of PDMS particles and the polystyrene particles (with acoustic contrast factor of 0.49, as a standard bead with positive contrast factor). The acoustic wave field creates a 2D patterned array of pressure nodes (dark area in Figure S8a, Supporting Information) and antinodes (red area in Figure S8a, Supporting Information), where the pressure nodes can only trap particles of positive contrast factor, and the antinodes can only trap particles of negative contrast factor, respectively. As shown in Figure S8b (Supporting Information), PDMS particles were trapped at the same horizontal or lateral positions as the polystyrene particles, indicating that the contrast factors of these two particles were both positive. This result proves the feasibility of PDMS particles to represent positive contrast factors.

The cornerstone of our device was a pair of patterned gold electrodes strategically deposited on a Y‐36 LiNbO_3_ substrate, yielding a thumb‐sized platform. While the 36° y‐cut lithium niobate (LiNbO_3_) substrate exhibits some anisotropy, its effect on our acoustography was negligible due to our design considerations. The acoustography pattern was primarily formed by leaky waves propagating directly from the interdigital transducer (IDT) electrodes into the liquid environment, rather than by surface acoustic waves on the substrate plane. Our IDT design concentrates acoustic energy from a 6 mm diameter electrode area into a 300 µm diameter acoustography pattern, resulting in signal intensities orders of magnitude higher than any potential background noise. This design, combined with the damping effect of wave propagation through water, effectively minimizes the influence of any waves generated on the substrate outside the IDT area. Consequently, the acoustic phase velocity differences for different directions on the substrate have minimal impact on both our holograph formation and IDT design, rendering the substrate's anisotropic properties largely inconsequential for our acoustography patterns. In all the experiments of 2D trapping and 3D tracing, as well as the simulation examples in this paper, it use a frequency of 22–30 MHz and an amplitude of 15 Vpp (2D patterning) or 30 Vpp (3D tracing) for the radio‐frequency signals added on the pair of electrodes. To perform experiments, a raised PDMS chamber with a side length of ≈10 mm was attached to the device's patterned side, covering the high‐intensity area. This chamber, once filled with the requisite liquid, was sealed with a thin PDMS lid, minimizing acoustic reflection. As water was used as the liquid environment for all the experimental setups, the speed of sound in the chamber was set to 1480 m s^−1^.

For both 2D trapping and 3D tracing experiments, the closed chamber configuration was employed by aligning its height to be ≈300 µm more than the focusing distance and positioning the pattern at the center of the interface. For 2D trapping, it was also optional to have an open chamber configuration. This design not only simplifies the process of introducing and retrieving samples but also enhances the efficiency of trapping or concentrating samples. As our focus primarily was to utilize heptane microdroplets in an aqueous medium, the chamber was positioned below the substrate on the patterned side. Given heptane's lower density compared to water, this arrangement leverages the buoyancy force to counterbalance the acoustic radiation force in the +z direction, mitigating repelling forces to particles from the trap.

### Microscopy Setup

The microscopic images of the 2D and 3D particle clusters were captured on the upright Olympus microscope system, BX51W1, and the OMAX A35180U3 camera. The Confocal experiment was carried out by the Andor Dragonfly Spinning Disk Confocal plus system.

### Preparation of Heptane Microdroplets

To clearly visualize and track patterned particles under microscopy and confocal imaging, 2D and 3D acoustic fields were generated using fluorescent heptane microdroplets in an aqueous medium. Crucial to this approach was incorporating surfactants to prevent droplets from remerging and a fluorescent dye (Ritchie Engineering Inc.) to provide a strong signal. Specifically, heptane (Sigma–Aldrich, Germany) was first mixed with the surfactants SPAN 80 (4% v/v, Sigma–Aldrich, Germany) and SDS (1% v/v, Sigma–Aldrich, Germany). This study then combine this surfactant‐infused oil with the oil‐soluble fluorescent dye Yellow Jacket (USA) at a 10:1 volume ratio. This fluorescent oil mixture was added to Milli‐Q water containing the surfactant BSA (10% v/v, Sigma–Aldrich, Germany), again using a 10:1 volume ratio. To generate droplets of 20–50 µm diameter, it briefly sonicate this emulsion for 1 s. The resulting fluorescent, surfactant‐stabilized heptane droplets enable clear observation and tracking of acoustically patterned particles.

### Preparation of DNA Carrier Droplets

Similarly, to generate DNA carrier droplets, it create stable microdroplets of silicon oil (Sigma–Aldrich, Germany) infused with the mineral oil (Sigma–Aldrich, Germany) at a 4:1 volume ratio. This stabilized oil was added to Milli‐Q water with BSA (10% v/v, Sigma–Aldrich, Germany) at a 10:1 volume ratio and briefly sonicated to form droplets of 20–50 µm diameter. These droplets were then mixed 1:1 with DNA oligos (IDT, USA) modified on one end with a hydrophobic moiety. This amphiphilic DNA spontaneously incorporates onto the water–oil interface to generate DNA‐infused oil microdroplets without additional fluorescent dyes. The resulting droplets enable fluorescent visualization for acoustic manipulation experiments.

### Device Fabrication

The fabrication process of our BEACON device begins with cleaning the Y‐36 LiNbO_3_ substrate. This process was first achieved through immersion in a piranha solution, followed by plasma wash to remove nanoscale contaminants and evaporate residual solvents from the substrate surface. With the substrate prepared, it proceed to lithography using a mask featuring the pattern of the two optimized electrodes. AZ1518 photoresist was utilized to ensure precise pattern transfer during the lithographic process. The depositing process involves depositing a 10 nm layer of Ti, followed by a 300 nm layer of gold, onto the substrate. Choosing a thicker gold layer minimizes Joule heating, a critical consideration for biomedical applications where temperature fluctuations could have detrimental effects. Subsequently, the photoresist lift‐off process was performed in acetone, yielding a clean and accurately patterned electrode structure.

A chamber using PDMS was fabricated with a height slightly larger than the focal distance designed to characterize the generated nodes. A 1:5 PDMS mixture of a silicone elastomer base and a curing agent was used. The height of the chamber was selected to allow the rapid formation of the entire 2D or 3D node near the top of the chamber. Meanwhile, the chamber dimensions in the *x*–*y* plane were carefully chosen to be large enough to mitigate reflection from the walls, ensuring accurate and undisturbed node characterization.

### Statistical Analysis

Most experiments in this work do not require statistical analysis as they demonstrate qualitative results or proof‐of‐concept demonstrations. For quantitative measurements in Figure 4c, mean ± standard deviation (SD) values were reported. Specifically, the average distance between adjacent high‐intensity regions was measured as 24.43 ± 5.55 µm (n = 20 measurements). The average width of each high‐intensity dot pattern in the lower concentration experiment was 13.29 ± 2.52 µm (n = 20 measurements). These measurements were made using ImageJ software (NIH) to analyze microscopy images. No statistical hypothesis testing was performed as these measurements were intended to characterize system resolution rather than test for significant differences between conditions.

### AI Usage

ChatGPT 4.0 was used to check the grammar.

## Conflict of Interest

T.J.H. has co‐founded a start‐up company, Ascent Bio‐Nano Technologies Inc., to commercialize technologies involving acoustofluidics and acoustic tweezers.

## Author Contributions

W.Y. conceived the idea, lead the experimental work, data analysis and scientific presentation. H.Z. contributed to the coding and polishing of figures. W.Y. and H.Z. contributed to the theory and data processing. W.Y., H.Z., and N.U. wrote the paper. B. L. contributed to the rendering of schematics. L.P.L. and T.J.H. provided overall guidance and contributed to the experimental design and scientific presentation.

## Supporting information



Supporting Information

Supplemental Movie 1

Supplemental Movie 2

Supplemental Movie 3

## Data Availability

The data that support the findings of this study are available from the corresponding author upon reasonable request.
