# Supplementary material for: Acoustography by Beam Engineering and Acoustic Control Node: BEACON
Source: Adv Sci (Weinh). 2024 Oct 18;11(46):2403742. doi: 10.1002/advs.202403742 (PMC11633508; doi:10.1002/advs.202403742)
Supplement: Supplementary file 1 — Supporting Information [file ADVS-11-2403742-s003.pdf]

ADVANCED SCIENCE
Open Access

Supporting Information

for *Adv. Sci.*, DOI 10.1002/advs.202403742

Acoustography by Beam Engineering and Acoustic Control Node: BEACON

Wenjun Yu, Haodong Zhu, Neil Upreti, Brandon Lu, Xianchen Xu, Luke P Lee and Tony Jun Huang**

Supporting Information

Acoustography by Beam Engineering and Acoustic Control Node: BEACON

Wenjun Yu, Haodong Zhu, Neil Upreti, Brandon Lu, Xianchen Xu, Luke P Lee, and Tony Jun Huang**

Wenjun Yu, Haodong Zhu
Department of Mechanical Engineering and Material Science, Duke University
NC, 27708, Durham, USA
Neil Upreti, Brandon Lu
Department of Biomedical Engineering, Duke University
NC, 27708, Durham, USA
Xianchen Xu
Department of Mechanical Engineering and Material Science, Duke University
NC, 27708, Durham, USA
Luke P Lee
Harvard Medical School, Division of Engineering in Medicine,
Department of Medicine, Brigham and Women's Hospital, Harvard University
MA, 02115, Boston, USA
Department of Bioengineering, Department of Electrical Engineering and Computer Science,
University of California, CA, 94720, Berkeley, Berkeley, USA
Institute of Quantum Biophysics, Department of Biophysics, Sungkyunkwan University,
Suwon, 16419, Korea
Email: lplee@bwh.harvard.edu
Tony Jun Huang
Department of Mechanical Engineering and Material Science, Duke University
NC, 27708, Durham, USA
Email: tony.huang@duke.edu

This file includes:

Supplementary Note 1.	Space–bandwidth product.
Supplementary Note 2.	Generation of IDT from the optimized phase diagram.
Supplementary Note 3.	The Gor’kov potential.
Supplementary Note 4.	Electrode spacing distribution and its relation to acoustic field properties.
Supplementary Note 5.	IDT layout generation process.
Figure S1.	Formation of 2D acoustography.
Figure S2.	Demonstration of BEACON's capability to create arbitrary patterns.
Figure S3.	Phase change along a 3D acoustography with the shape of the singular helix with negative and positive m, respectively.
Figure S4.	Demonstration of generation of IDT from the phase diagram.
Figure S5.	Optimization of IDT thresholding.
Figure S6.	Example of generated IDT electrode patterns used in this work, for 2D rectangle and triple helix, respectively.
Figure S7.	Relative acoustic intensity profile displayed in this work.
Figure S8.	Comparison of the use of initial phase diagram with random phase initialization method in the Wirtinger hologram algorithm.
Figure S9.	2D acoustic patterning of PDMS and polystyrene microparticles.
Figure S10.	Demonstration of the setup of BEACON.
Figure S11.	Process of generating the IDT layout from an optimized phase diagram.
Figure S12.	Demonstration of the impact of unconnected regions in IDT design.
References	*35, 46, 51, 54, 55, 57, 58*
Supplementary Movie 1.	Rapid formation of 2D pattern with heptane droplets.
Supplementary Movie 2.	Circulating polystyrene particles and heptane droplets in the same ring-shaped acoustography.
Supplementary Movie 3.	Particle tracing within the triple helix with z gradually increasing from 0 to f.
Table S1.	Pseudo-code for Wirtinger hologram algorithm.

Supplementary Note 1. Space-bandwidth product

Generally, the content of information of a wavefront is defined by the scalar space–bandwidth product (SW). A Larger value of SW can lead to a larger area for patterning and a finer reconstruction of the acoustography. The SW of an interdigital transducer (IDT) is defined as N^2, where N is the number of pixels of the IDT image in either the x or y direction. As there is an inevitable information loss during the wave propagation, and the amount of information of the acoustography cannot exceed the diffraction limit, this SW is further limited by[35]

$$\mathrm{SW}_{max} = 4D^2/\lambda^2 \tag{S1}$$

where D is the diameter of the IDT (or the scale of the hologram as in Figure 1d), and λ is the wavelength.

To calculate the pixel size in our BEACON system, we consider both theoretical and practical limitations:

Theoretical limit: The minimum pixel size is typically limited to half the wavelength (λ/2) to avoid aliasing effects.

Practical limit: This is determined by the aperture of the system, calculated as d = L / N, where L is the size of the acoustography in one axis, and N is the number of the smallest distinguishable units in one axis.

The larger value between the theoretical and practical limits is taken as the effective pixel size. Our practical limit is smaller due to our high space-bandwidth product and large effective aperture size relative to the wavelength.

Supplementary Note 2. Generation of IDT from the Optimized Phase Diagram

Our device features patterned electrodes on a piezoelectric substrate, harnessing its inherent properties to generate acoustic waves upon signal application. Our IDT employs a dual-electrode design, with each electrode activated by a unique signal and a phase shift of π between these signals to ensure mutual reference (Figure S4, a and b). To create a coherent acoustography pattern, our design methodology focuses on precisely patterning two isophase patterns on the substrate, each corresponding to one of the electrodes. This process entails the careful construction of coherent patterns for each electrode, as well as considering various factors, such as the electrode spacing and geometry, which influence the resulting acoustic field (Figure S5). For each IDT generated, we apply two different thresholds on the phase diagram generated from the Wirtinger hologram algorithm with a phase difference of π as the pattern of each electrode. Following a

similar process as shown in Figure S5, we optimize the acoustography generated by each pattern by refining the initial phase and threshold value selected to minimize the MSE loss of the node generated compared with the target. Finally, the pair of electrodes is added (as shown by the pair of continuous curved lines in Figure S4c), and the thresholded pattern is slightly modified based on the position of electrodes to prevent any potential short or open circuits.

Supplementary Note 3. The Gor'kov potential

Considering the interaction of microdroplets with the acoustography generated, the force added on the droplet is defined by the gradient of Gor'kov potential:[54]

$$F = -\nabla U_G \tag{S2}$$

$$U_G = C_1|p|^2 + C_2\left(|p_x|^2 + \left|p_y\right|^2 + |p_z|^2\right) \tag{S3}$$

$$C_1 = \frac{1}{4}(\beta_0 - \beta_P) \tag{S4}$$

$$C_2 = \frac{3}{4}V\left(\frac{\rho_0 - \rho_P}{\omega^2\rho_0(\rho_0 + 2\rho_P)}\right) \tag{S5}$$

where p is the complex acoustic pressure generated, V is the volume of the droplet, ω is the frequency of the emitted acoustic waves, ρ is the density, and $\beta = 1/\rho c^2$ is the compressibility (with c referring to the speed of sound, and the subscripts 0 and P referring to the host medium and the particle material, respectively). Following the previous results of force analysis on a small spherical particle in Bessel Beam,[57-58] we can estimate the acoustic contrast factor for particles used in this work as

$$\emptyset_P = \frac{\beta_0 - \beta_P}{2\beta_0} + \frac{3(\rho_P - \rho_0)}{4(\rho_0 + 2\rho_P)} \tag{S6}$$

Supplementary Note 4: Electrode spacing distribution and its relation to acoustic field properties

The electrode spacing in our design corresponds to the entire acoustography shape, rather than just specific portions. This comprehensive patterning ensures uniform phase distribution across the whole acoustography. The observed variation in spacing—wider in the middle and narrower towards the edges—stems from the phase distribution characteristics of a spherical vortex point source, as described by Baudoin et al. (2019).[46] When projected onto a flat surface, this naturally

results in a pattern where the spacing is broader near the center and tighter at the edges, due to the steeper change of radius in the phase diagram pattern near the center. Our approach, which integrates point-based Bessel beam foci along predetermined trajectories, maintains this characteristic spacing trend across various designs. Importantly, while local electrode spacing is crucial for phase modulation, it does not directly influence the intensity distribution of the acoustic field. The acoustic amplitude is predominantly governed by the overall electrode pattern rather than variations in local spacing.

Supplementary Note 5: IDT Layout generation process

The process of generating the IDT layout from the optimized phase diagram involves several steps, as illustrated in Figure S11, which include:

1) Phase Thresholding: We begin by applying phase thresholding to the final continuous phase distribution ready for fabrication. This generates two electrode patterns corresponding to phases near 0 and π, respectively (Figure S11a). The regions for the two electrodes are marked in blue and purple.
2) Manual Refinement: We then manually refine the electrode layouts. The dash-dotted lines in both Figure S11a and S11b delineate the regions where we will place bus lines connecting all spiral structures for both the blue (frame #1) and purple (frame #2) electrodes. As illustrated in the magnified view in Figure S11b, the addition of these bus lines intersects some patterns from the opposing electrode, potentially creating isolated segments. For instance, the purple electrode region in Figure S11a near frame #3 would become isolated when we add a blue bus line in frame #1. To address this isolation, we manually connect these isolated structures with thin lines in areas such as those marked by frames #3, #4, and #5, ensuring that all regions of the same color remain interconnected as a single electrode.
3) Final Layout: With these modifications, we obtain the complete IDT layout, as shown in Figure S11c. The solid line wireframes in Figures S11b and S11c show how the bus lines extend beyond the IDT from points #6 and #7, connecting to millimeter-scale contact pads for each electrode. These pads can be connected to different electric actuation signals, allowing for the necessary phase shift between the two electrodes.

In cases where phase patterns aren't connected via continuous traces, we exclude these regions from the IDT design. Figure S12 demonstrates the minimal impact of this approach. Images i and ii show electrode patterns with and without the central part (containing small unconnected regions), while images iii and iv display the corresponding acoustic intensity patterns. The negligible difference between these patterns confirms that omitting small unconnected regions does not significantly affect the acoustography quality.

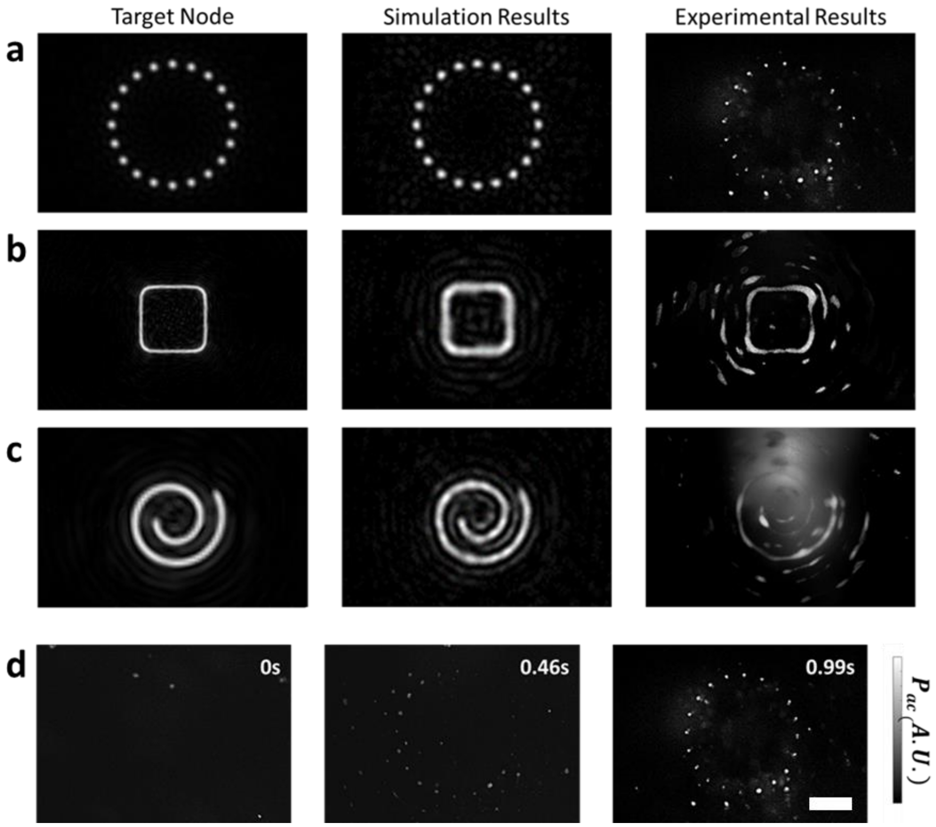

Figure S1. Formation of 2D acoustography. a-c) The acoustography target (first column), corresponding simulation results (second column), and experimental results (third column) of the (a) circle of dots, (b) square, and (c) spiral shape, respectively. d) Time-lapsed images recording the formation process of the circle of dots. Scale bar is 120 μm.

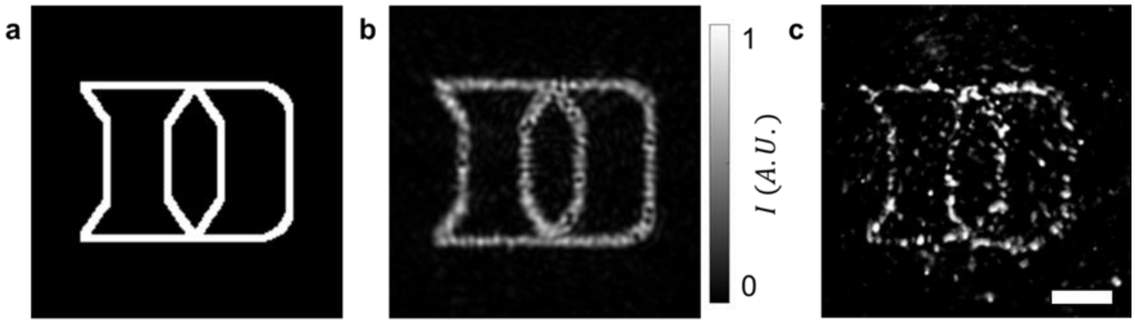

Figure S2. Demonstration of BEACON's capability to create arbitrary patterns. a) A target pattern presented as the Duke sports logo. b) A simulation result showing the intensity distribution generated by the optimized acoustography. c) An experimental result displayed as an image of heptane microdroplets trapped in the pattern of this letter logo (scale bar: 200 μm).

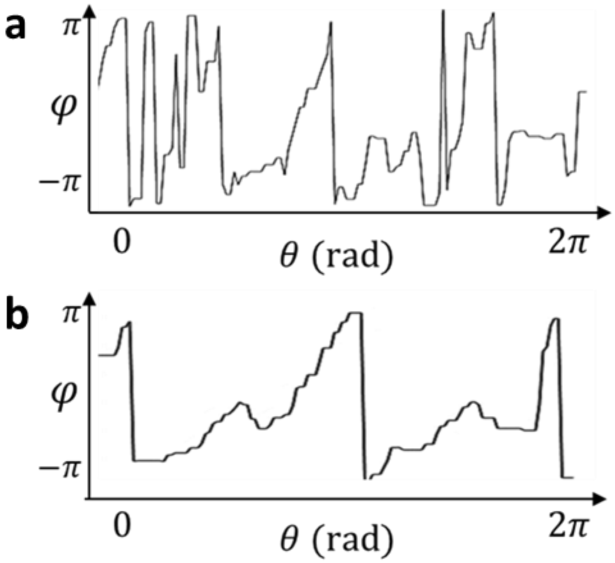

Figure S3. Phase change along a 3D acoustography with the shape of a singular helix with a) negative and b) positive m, respectively. Analysis of the phase distribution along a single helix, emphasizing the necessity of a uniform phase distribution to successfully realize complex 3D structures.

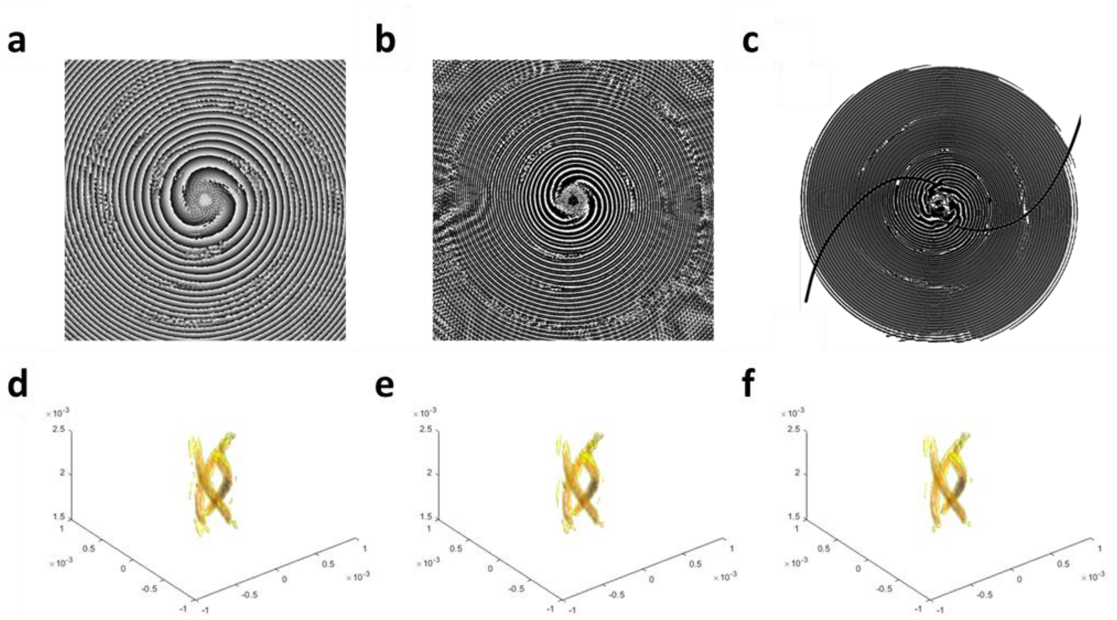

Figure S4. Demonstration of generation of IDT from the phase diagram. a-c) Example phase diagram generated from (a) the Wirtinger hologram algorithm, and (b) the corresponding thresholded image and (c) the IDT electrode pattern developed accordingly. d-f) The colored threshold of acoustic intensity generated from (d) the phase diagram, (e) the thresholded image, and (f) the final IDT pattern, respectively. Orange threshold: 50% max intensity; yellow threshold: 25% max intensity.

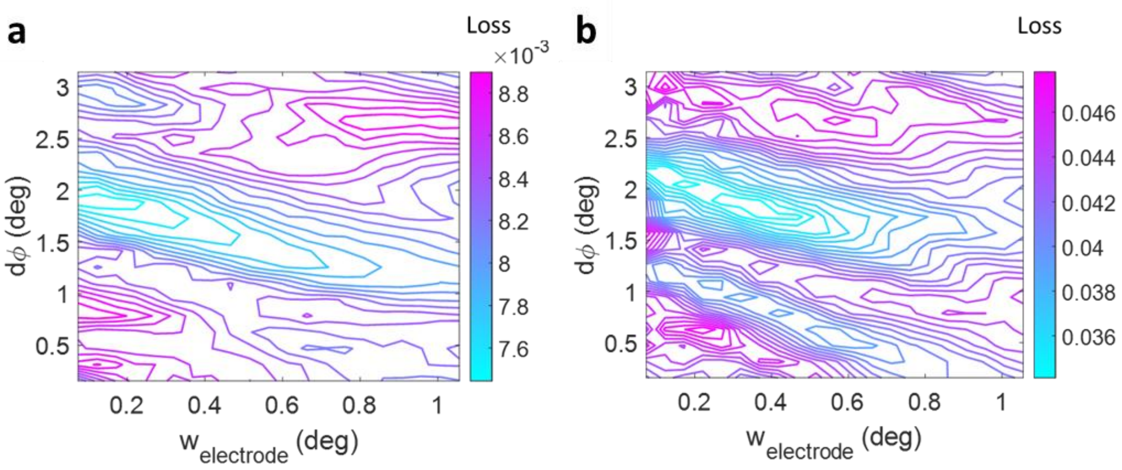

Figure S5. Optimization of IDT thresholding. a, b) The MSE loss versus the threshold value selected (*x*-axis in the plot) and initial phase shift (*y*-axis in the plot) with an example (a) 2D and (b) 3D autograph pattern, respectively.

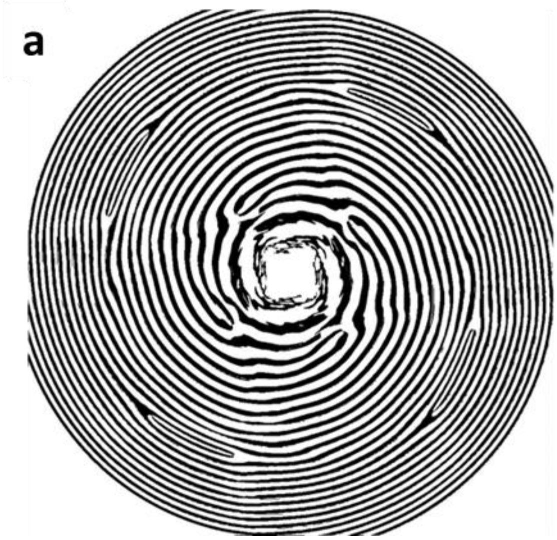

Figure S6. Example of generated IDT electrode patterns used in this work, for a) 2D rectangle and b) triple helix, respectively.

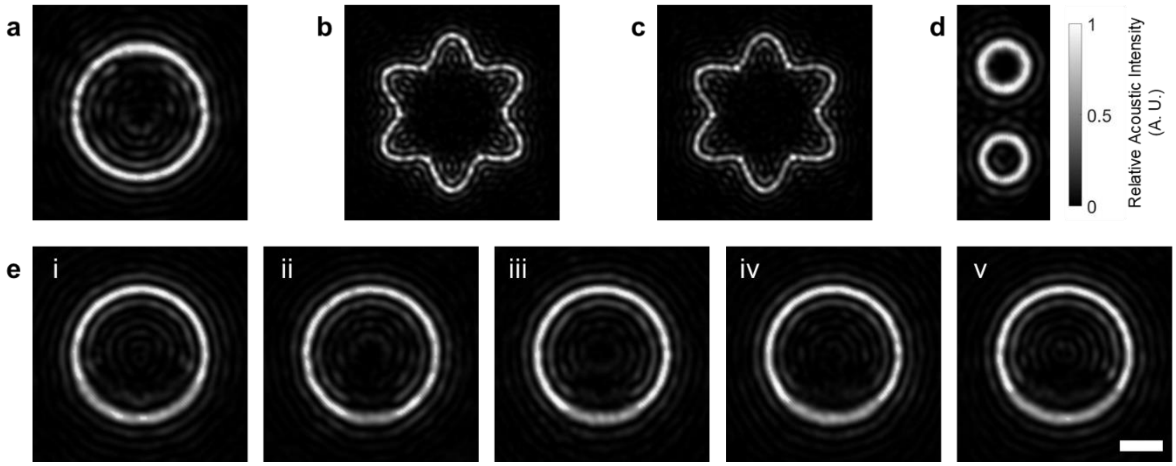

Figure S7. Relative acoustic intensity profile displayed in this work, for a) Figure 1e, b-c) Figure 2c, (i-ii), d) Figure 4e, e) Figure 4g, (i-v), respectively. Scale bar is 200 μm.

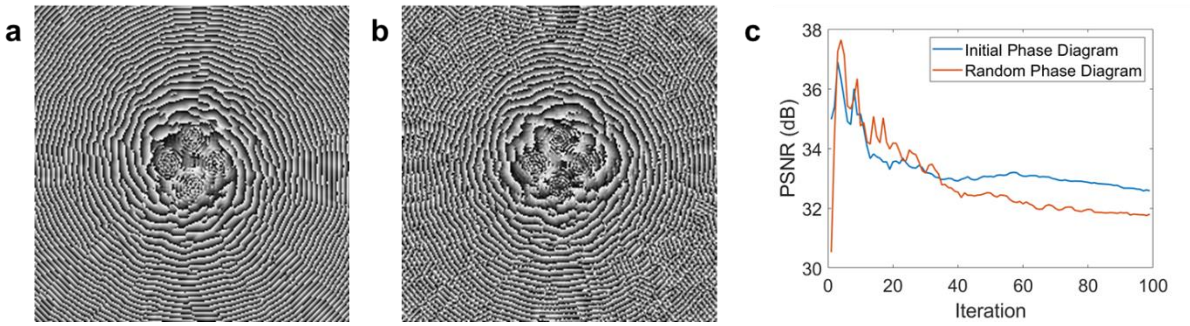

Figure S8. Comparison of a) the use of the initial phase diagram with b) the random phase initialization method in the Wirtinger hologram algorithm. The same target node is applied to (a) and (b), and the output is collected after 100 iterations. A continuous phase diagram (especially for the area far from the center of the figure) can ensure the quality of the electrode in the thresholding process (Supplementary Note 3) and thus improve the final result [51]. c) The change of peak signal-to-noise ratio in (a-b) during the iteration process. Using an initial phase diagram leads to a higher PSNR than the random phase initialization method starting from iteration 40.

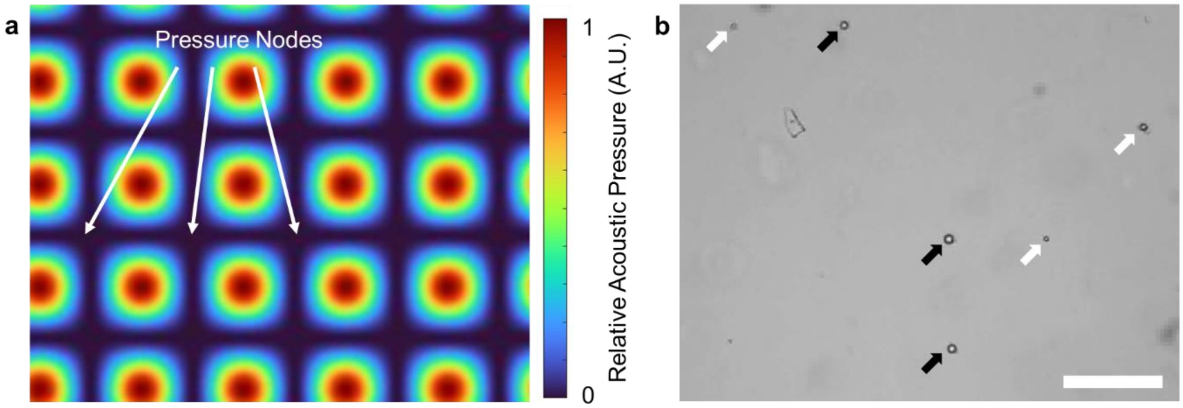

Figure S9. 2D acoustic patterning of PDMS and polystyrene microparticles. a) The relative acoustic pressure field for 2D patterning. The acoustic wave field creates a 2D patterned array of pressure nodes (dark area) and antinodes (red area), where the pressure nodes can only trap particles of positive contrast factor, and the antinodes can only trap particles of negative contrast factor, respectively. Each pressure node has a distance of 100 μm to adjacent nodes in both directions, and the position of the pressure nodes and antinodes matches the position in (b). b) Experimental result for particle patterning. The black arrows indicate the polystyrene microparticles (with a diameter of 10.29 μm), and the white arrows indicate the PDMS microparticles. The PDMS particles are trapped at the same horizontal or lateral positions as the polystyrene particles, indicating that the contrast factors of these two particles are both positive. Scale bar is 100 μm.

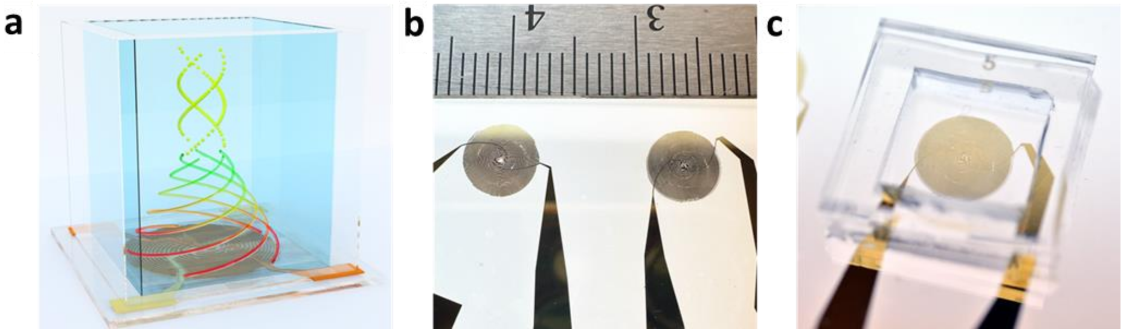

Figure S10. Demonstration of the setup of BEACON. a) Schematic demonstration of the experiment process. b) Photo of a real device fabricated compared with actual scaling. c) Photo of the experimental setup with a PDMS chamber over the device.

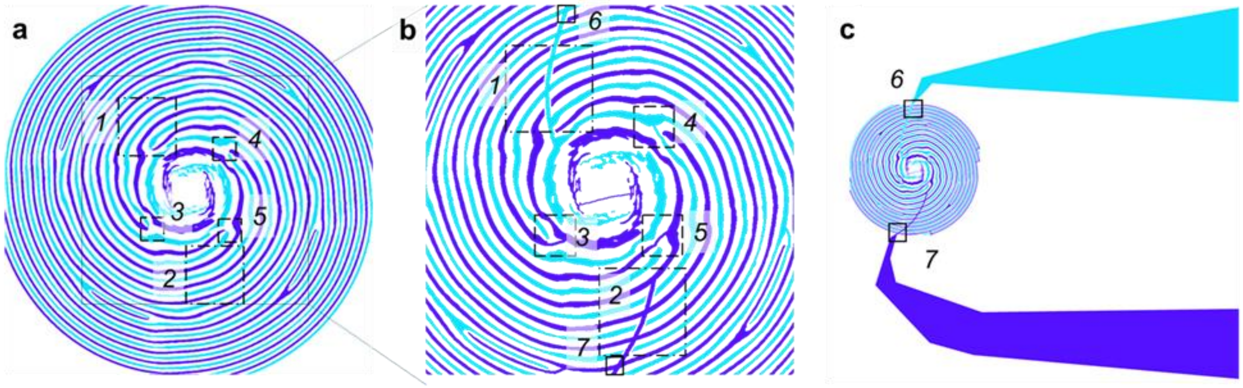

Figure S11. Process of generating the IDT layout from an optimized phase diagram. a) Result after phase thresholding of the optimized phase diagram for two electrodes near phases 0 and π. b) Magnified view of the central region of one electrode, showing the manual design of electrode positions. c) Complete IDT layout with two interconnected electrodes shown in light and dark blue, including large electrodes representing bus lines for different electric actuation signals.

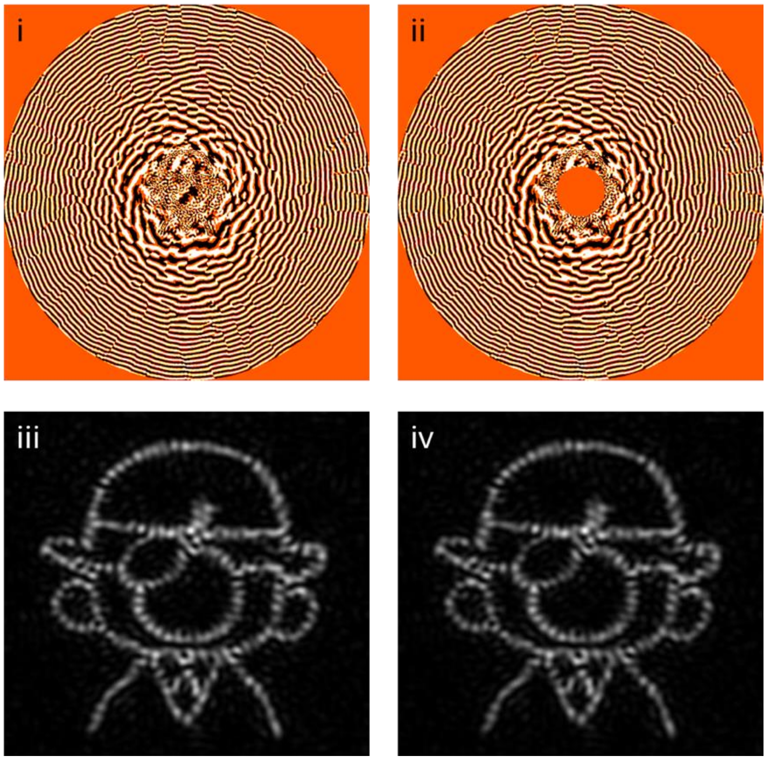

Figure S12. Demonstration of the impact of unconnected regions in IDT design. i) Electrode pattern including all regions. ii) Electrode pattern with central part (containing small unconnected regions) removed. iii) Acoustic intensity pattern corresponding to the full electrode design in (i). iv) Acoustic intensity pattern corresponding to the modified electrode design in (ii). The negligible difference between (iii) and (iv) demonstrates that omitting small unconnected regions does not significantly affect acoustography quality.

Table S1: Pseudo-code for Wirtinger hologram algorithm

Input:
Target shape function $\xi(t)$;
focusing distance f;
propagation function $\mathcal{H}$;
error function $Err(I, I_{target})$;

Output:
Optimal phase diagram Φ_{out}

for k in range $(0, n)$:

$I_{target}(:,:,k) = |\mathcal{H}(\mathcal{L}\{B(\xi(t))\}, dz(2k-n)/2n)|^2$

end

$\Phi_0 = arg(\mathcal{H}(B_0, -f))$

// Generate the target intensity distribution and the initial phase diagram based on Equation (1-5)

---

while *not converged*:

for k in range $(0, n)$:

$P = \mathcal{H}(\Phi, f + dz(2k-n)/2n)$ // Compute reconstructed fields

$I = |P|^2$ // Compute reconstructed intensity

$Err_k = g\left(|P|^2, I_{target}(:,:,k)\right) = g(P)$

$Err = Err + \frac{1}{N^2}\sum_{i=1}^{N}\sum_{j=1}^{N}\left[I(i,j,k) - I_{target}(i,j,k)\right]^2$ // Compute loss function as a sum-up of each layer in *z* direction

$\partial\Phi_\mathrm{k} = \mathcal{H}(\nabla g, -f - dz(2k-n)/2n)$

$\nabla g = 2\nabla_{\bar{P}} g = 2(I(:,:,k) - I_{target}(:,:,k)) \circ 2P$ // Compute Wirtinger gradient

$\partial\Phi = \partial\Phi + r_k\, \partial\Phi_\mathrm{k}$ (∘ is defined as the element-wise multiplication)

end

$\Phi = \mathrm{L\_BFGS}(\Phi, \partial\Phi)$ // Step-based optimization via the L-BFGS algorithm[55]

end

---

return $\Phi_\mathrm{out} = \Phi$
